# Multimodal AI in high-grade serous ovarian cancer: integrated prediction and clinical decision-making

**DOI:** 10.3389/fonc.2026.1874918

**Published:** 2026-07-31

**Authors:** Marie Pelissier-Combescure, Jean-Philippe Villemin, Damien Bonzom, Charles Berger, Chad Estoup-Streiff, Yulia Lakhman, Ramona Woitek, Pierre-Emmanuel Colombo, Zijing Lin, Haiming Li, Stéphanie Nougaret

**Affiliations:** 1 Precision Imaging as a New Key in Cancer Care (PINKCC) Lab, Montpellier Cancer Research Institute (IRCM), Univ Montpellier, Inserm, ICM, Montpellier, France; 2 Department of Radiology, Memorial Sloan Kettering Cancer Center, New York, NY, United States; 3 Research Center for Medical Image Analysis and Artificial Intelligence, Department of Medicine, Faculty of Medicine and Dentistry, Danube Private University, Krems an der Donau, Austria; 4 Montpellier Cancer Institute, Department of Surgery, Montpellier, France; 5 Fudan University Shanghai Cancer Center, Department of Radiology, Shanghai, China; 6 Montpellier Cancer Institute, Department of Radiology, Montpellier, France

**Keywords:** artificial intelligence, deep learning, image processing, integrative omics, machine learning, multimodal imaging, multiomics, ovarian neoplasms

## Abstract

Ovarian cancer remains the most lethal gynecologic malignancy, with high-grade serous ovarian carcinoma (HGSOC) accounting for the majority of deaths. It is typically diagnosed at an advanced stage, characterized by extensive peritoneal dissemination and high rates of recurrence, which contribute to persistently poor survival despite advances in cytoreductive surgery, platinum-based chemotherapy, and targeted therapies such as PARP inhibitors. Clinical outcomes are highly variable, reflecting substantial inter- and intratumoral heterogeneity at the genomic, cellular, and microenvironmental levels. Accurate survival prediction and treatment response assessment therefore remain major challenges. Current clinical and radiological evaluation tools provide only limited resolution of this complexity and fail to fully capture the spatial and temporal dynamics of the disease. As a result, patient stratification and treatment decision-making are often suboptimal, particularly in the context of evolving therapeutic strategies. Artificial intelligence (AI) has emerged as a powerful approach to extract clinically relevant information from diverse data sources, including CT and MRI radiomics, histopathology whole-slide images, and multi-omics data. While promising results have been reported within individual modalities, each captures only a partial view of the disease. Integrating these complementary data sources through multimodal AI approaches offers the potential to more comprehensively model tumor biology and improve predictive performance. In this review, we analyze 31 HGSOC studies using a unified multimodal pipeline taxonomy structured into five sequential stages: feature extraction, intra-modal aggregation, fusion strategy, inter-modal integration, and prediction head. For each stage, we identify and categorize the technical approaches employed across studies, highlighting both common design patterns and novel methodological contributions. To complement this analysis, we provide visual overviews, a paper-by-paper comparative visualization, and structured summary tables. We further discuss key challenges in the field, including inconsistent validation strategies, limited reproducibility, heterogeneous integration of genomic data, and insufficient attention to model explainability.

## 1 Introduction

Ovarian cancer remains the most lethal gynecologic malignancy, with high-grade serous ovarian carcinoma (HGSOC) accounting for the majority of deaths and a 5-year overall survival decreasing from 92% in early-stage to only 29% in advanced disease (1). HGSOC is now widely recognized not as a uniform entity but as a highly heterogeneous and evolving ecosystem, characterized by extensive genomic instability, subclonal diversity, and dynamic interactions with the tumor microenvironment (2, 3).

This heterogeneity spans multiple biological scales, including tumor cell clones, stromal and immune compartments, epigenetic regulation, and metabolic states (4–6). Single-cell and spatial transcriptomic studies have revealed the coexistence of multiple tumor subclones within the same lesion, each with distinct transcriptional programs and copy-number alterations, as well as diverse microenvironmental niches composed of fibroblasts, immune, endothelial, and mesothelial cells. These components interact through spatially organized signaling networks, including ligand–receptor and autocrine loops, shaping local tumor behavior and therapeutic response (4, 7).

Importantly, this biological complexity is tightly linked to treatment resistance. Increased tumor cell state heterogeneity and functional clonality have been associated with poor prognosis and chemotherapy resistance, while specific microenvironmental features, such as CXCL12^+^, cancer-associated fibroblasts correlate with platinum resistance and adverse outcomes (8–13). Aggressive epithelial subpopulations characterized by high proliferative and migratory potential, as well as immunosuppressive or heterogeneous immune landscapes, further contribute to resistance to both platinum-based chemotherapy and poly (ADP-ribose) polymerase (PARP) inhibitors (9, 14, 15).

As a consequence, accurate survival prediction and treatment response assessment remain major challenges. Conventional biomarkers and clinical tools, including CA-125, standard imaging, and clinicopathological variables, capture only limited aspects of this complexity. CA-125 lacks specificity and can be elevated in many benign and non-gynecologic conditions, and its diagnostic sensitivity is limited, especially in early disease, leading to high false-positive and false-negative rates (16, 17). A substantial subset of patients has normal or only borderline CA-125 elevations, and even in follow-up some recurrences are detected radiologically or symptomatically without CA-125 rise, underscoring that CA-125 alone is an imperfect marker for diagnosis, surveillance, and prognosis (1). Single biopsies or conventional imaging snapshots also fail to reflect the pronounced spatial and temporal heterogeneity of HGSOC: multi-site and longitudinal sampling shows divergent subclones and copy-number patterns across sites and over time, with clonal expansion metrics correlating with worse PFS and OS (18, 19). Non-invasive imaging modalities, including computed tomography (CT), positron emission tomography (PET/CT), magnetic resonance imaging (MRI) and ultrasounds (US), are essential for disease staging and monitoring but primarily capture macroscopic tumor burden. Radiological response classifications frequently show discordance with underlying tumor biology and serum biomarkers such as CA-125, and independent central review has been shown to reclassify response categories in a substantial proportion of cases. These findings highlight the limitations of imaging as a standalone tool for prognostic assessment and treatment response evaluation (20–23). Prognosis instead reflects a complex integration of genomic alterations (e.g., BRCA/HRD status), pathway activation, epigenetic states, and tumor-microenvironment interactions, none of which can be adequately represented by a single modality.

Early artificial intelligence (AI)-based approaches in HGSOC have predominantly relied on unimodality including computed tomography and magnetic resonance imaging radiomics, histopathology whole-slide images (WSIs), and clinical variables. While these unimodal approaches have achieved notable performance, no single modality can adequately reflect the multi-scale complexity of HGSOC biology.

In contrast, multimodal integration offers a more comprehensive framework by combining complementary information across biological scales, from macroscopic tumor burden in radiology to tissue architecture in histopathology and molecular mechanisms captured by omics data. In this context, “multimodal approaches” refer to methods that enable the integration of different types of modalities: imaging-based modalities, such as radiology (CT, MRI, PET) and histopathology, structured clinical data, as well as multi-omic data, including genomic, transcriptomic, and proteomic measurements, each of which is treated as a fully independent modality in its own right. This integrative approach has consistently demonstrated improved predictive performance compared with unimodal models (24–27). Beyond performance gains, multimodal AI directly addresses key clinical challenges, including preoperative prediction of platinum resistance, assessment of surgical resectability, and decision support in pathology, particularly important in a disease where invasive sampling is often limited or contraindicated (24, 28).

Despite these advances, existing reviews on AI in ovarian cancer (29–32) largely focus on unimodal approaches or provide high-level overviews of multimodal integration. They rarely offer a structured dissection of multimodal pipelines or detailed technical taxonomies of the methods employed at each stage, limiting their utility for researchers aiming to design or reproduce such models.

**Figure 1:**
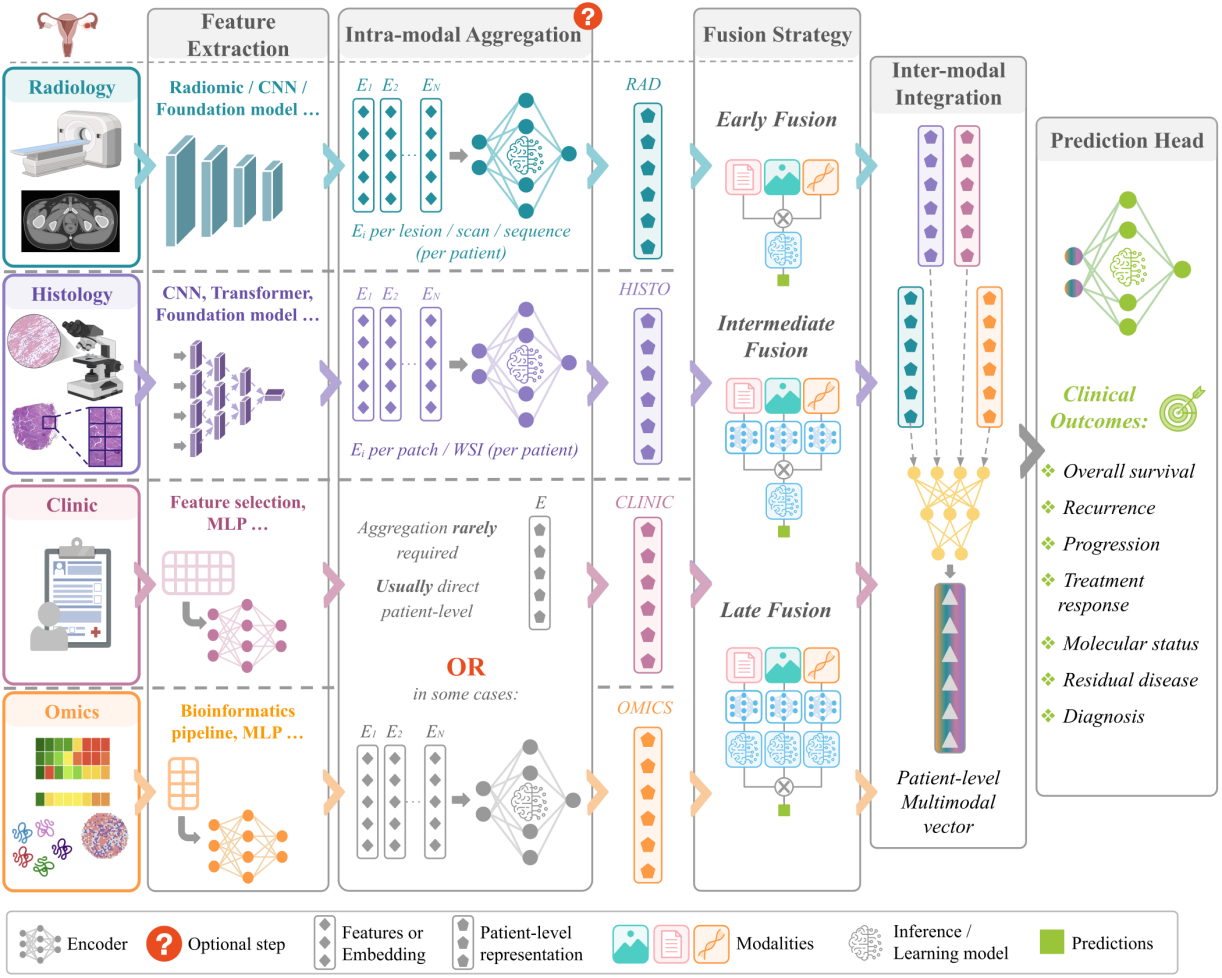
Overview of the five sequential stages in a unified multimodal pipeline taxonomy. Data modalities considered as inputs include radiology, histology, clinical variables, and omics profiles. These modalities are processed through five consecutive stages (1). Feature Extraction: each modality is transformed into a shared scalar feature space using modality-specific algorithms, ensuring the extraction of relevant representations. Depending on the approach, this stage produces either interpretable features (e.g., hand-crafted radiomic descriptors or selected clinical variables) or non-interpretable embeddings (e.g., deep features extracted by AI-based models such as MLPs, CNNs, Transformers, or foundation models, among others) (2). Intra-modal Aggregation (optional step, indicated by the red question mark): when multiple features or embeddings are available within a modality (e.g., per lesion, scan, sequence, patch, or WSI), they are combined at the local level into a patient-level representation using strategies. For clinical and omics data, which are typically already structured at the patient level, this step is rarely necessary (3). Fusion Strategy: depending on when the modalities are combined within the pipeline, one of three paradigms can be applied, namely Early Fusion, Intermediate Fusion, or Late Fusion (4). Inter-modal Integration: the individual patient-level representations from each modality are fused together through integration mechanisms to produce a single patient-level multimodal vector (5). Prediction Head: this representation is passed to a predictive model to generate results adapted to the clinical task of interest, including overall survival, recurrence, progression, treatment response, residual disease, molecular status, or diagnosis.

**Figure 2:**
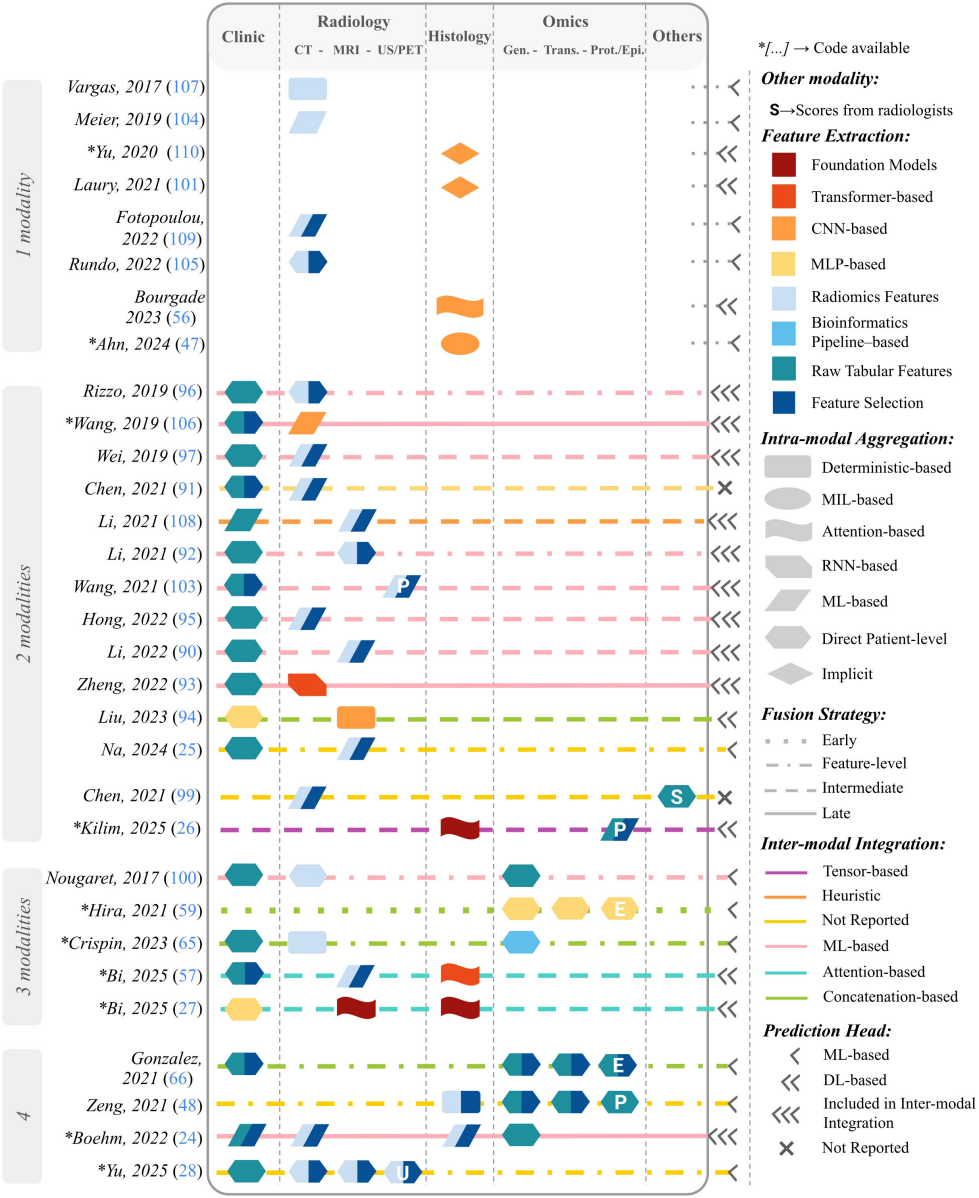
Sub-taxonomies of the five sequential pipeline stages. Each row corresponds to one reviewed study (referenced by first author and year), grouped vertically by the number of modalities used. Columns represent the input modalities considered, spanning clinical variables, radiology (CT, MRI, US/PET), histology (WSI), omics (genomics, transcriptomics, proteomics/epigenomics), and other sources. For each study, the algorithmic choices at each pipeline stage are encoded as follows (1): Feature Extraction is represented by the color of the shapes (2); Intra-modal Aggregation by the shape geometry (3); Fusion Strategy by the line style connecting modality-specific representations (4); Inter-modal Integration by the line color; and (5) Prediction Head by the arrow symbols at the end of each row. The complete legend detailing each encoding is provided alongside the figure. An asterisk (*) before the study name indicates that the source code is publicly available.

**Figure 3:**
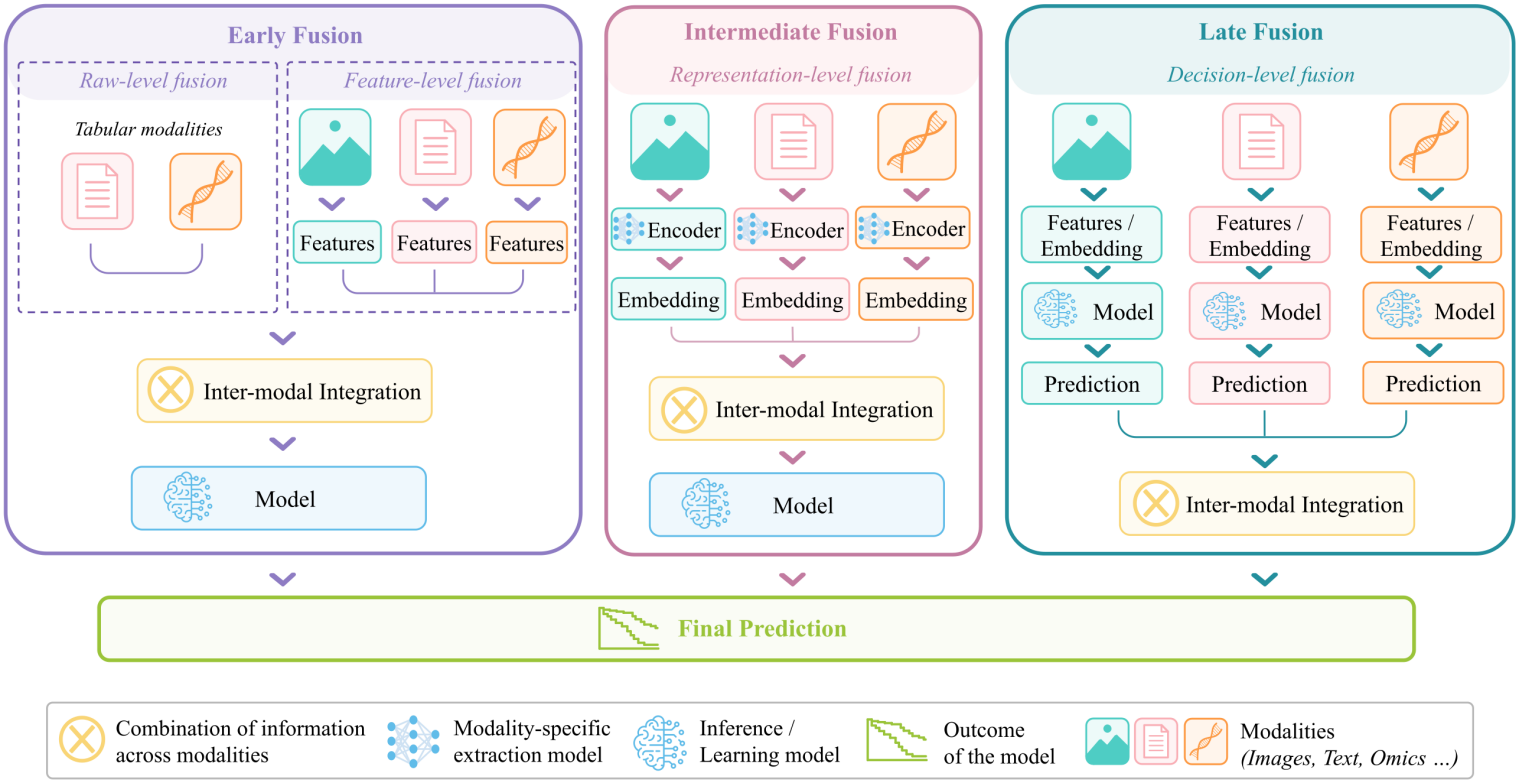
Overview of the multimodal fusion strategies. Main fusion paradigms are distinguished based on the stage at which modalities are combined within the pipeline. Early Fusion combines modality-specific information prior to any joint modeling. It encompasses two subtypes: *raw-level fusion*, which directly concatenates tabular modalities without prior encoding, and *feature-level fusion*, which first extracts modality-specific features independently before concatenating them as input to a shared model. Intermediate Fusion (representation-level fusion) first encodes each modality independently through dedicated modality-specific encoders to produce latent representations such as embedding, which are then integrated via an inter-modal integration mechanism before being passed to a joint predictive model. Late Fusion (decision-level fusion) trains independent modality-specific models, each generating its own prediction from the corresponding modality’s features or embeddings, and combines these individual predictions through an inter-modal integration step to produce the final output. All three strategies converge toward a single final prediction.

To address this gap, we analyze 31 HGSOC studies through a unified multimodal pipeline taxonomy comprising five sequential stages: feature extraction, intra-modal aggregation, fusion strategy, inter-modal integration, and prediction head. Importantly, the purpose of the proposed taxonomy is not to establish direct quantitative comparability between studies with heterogeneous datasets, clinical tasks, modalities, and validation schemes. Instead, it provides a unified conceptual framework to describe and analyze multimodal pipelines through common methodological stages, enabling structured interpretation of otherwise highly heterogeneous approaches. The 31 studies reviewed were selected based on four criteria: publication in peer-reviewed journals or conferences indexed in recognized quality lists according to our institution, exclusive focus on HGSOC, use of machine learning and/or deep learning models; and integration of at least two data modalities; eight unimodal studies were retained as a comparative baseline. **Figure 1** provides a general overview of the five-stage taxonomy pipeline, while **Figure 2** presents a paper-by-paper comparative visualization of these components across the 31 reviewed studies. **Figure 3** illustrates the diversity of fusion strategies. In addition, **Table 1** categorizes the reviewed studies according to their clinical task while **Tables 2**–**5** summarize dataset characteristics, cohort sizes, and validation strategies based on the number of modalities used in each study. We further identify key methodological limitations across studies, including inconsistent validation practices, limited reproducibility, and insufficient attention to model explainability.

**Table 1:** Clinical tasks addressed by the reviewed studies.

CLINICAL TASK	STUDIES
Overall survival prediction (OS)	(26, 48, 58, 92, 94, 106)
Recurrence prediction (RFS/DFS)	(89, 90, 93, 96, 105, 107)
Progression prediction (PFS)	(95, 99, 102)
OS and PFS combined prediction	(24, 27, 103, 108)
Residual disease/Operability prediction	(91, 95, 106, 108)
Treatment response prediction (Platinum/NACT)	(25, 26, 47, 57, 64, 65, 100, 104, 109)
Molecular status/BRCA prediction	(48, 56, 58, 99, 103)
Diagnosis/Staging/Lymph node metastasis	(28, 98)

**Table 2:** Training and validation cohorts for unimodal studies (1 modality).

		TRAINING				VALIDATION					
Study	Modalities	Country	Dataset	Centers	Cohort	Type	Country	Dataset	Centers	Cohort	Metrics
(106)	R	USA	Memorial Sloan Kettering Cancer Center	Private/Mono.	38	N/A	N/A	N/A	N/A	N/A	KM, log-rank p-value, Acc, TPR, TNR
(103)	R	USA	Memorial Sloan Kettering Cancer Center	Private/Mono.	88	N/A	N/A	N/A	N/A	N/A	KM, log-rank p-value, median, IQR
(109)	H	USA	TCGA-OV	Public/Multi.	456	Int.	-	-	-	114	AUC, KM, log-rank p-value, rs
(100)	H	Finland	Helsinki University Hospital	Private/Mono.	30	Int.	-	-	-	22	Acc, Se, Sp
(108)	R	Germany	Kliniken Essen-Mitte	Private/Mono.	323	Ext.	UK	Hammersmith Hospital ◦ TCGA	Private/Mono. ◦ Public/Multi.	224 ◦ NR	KM, log-rank p-value, HR, OR
(104)	R	UK	Cambridge University Hospitals	Private/Mono.	61	Ext.	UK	Barts Health	Private/Mono.	48	AUC, G-mean, Se, Sp, PPV, NPV
(56)	H	France	OvarIA	Private/Multi.	867	Ext.	USA	TCGA	Public/Multi.	NR	AUC, Dice score, IoU
(47)	H	Korea	Yonsei Severance Hospital	Private/Mono.	394	Ext.	Korea ◦ USA	Samsung Medical Center ◦ TCGA-OV	Private/Mono. ◦ Public/Multi.	136 284	AUC-ROC, precision, recall, Fl-score, KM, log-rank p-value

Center type - Mono., monocentric; Multi., multicentric. Modalities - R, Radiology; C, Clinical; H, Histology; O, Omics. Validation type — Int., internal; Ext., external. Cohort — N/A, no validation cohort reported; -, internal validation split; NR, Not Reported.

**Table 3:** Training and validation cohorts for multimodal studies (2 modalities).

TRAINING						VALIDATION					
Study	Modalities	Country	Dataset	Centers	Cohort	Type	Country	Dataset	Centers	Cohort	Metrics
(95)	C - R	Italy	European Institute of Oncology	Private/Mono.	101	N/A	N/A	N/A	N/A	N/A	Chi-square test, OR, AUC, DeLong test, VIF
(105)	C - R	China	West China Second University Hospital	Private/Mono.	151	Int. ◦ Ext.	- China	- ◦ Henan Provincial People’s Hospital	— ◦ Private/Mono.	49 ◦ 45	C-index, HR, AUC, Acc, HL test, DCA, KM, log-rank p-value, DeLong test
(96)	C - R	China	West China Second University Hospital	Private/Mono.	50	Int. Ext.	- ◦ China	- ◦ Henan Provincial People’s Hospital	- ◦ Private/Mono.	50 ◦ 42	C-index, Acc, log-rank p-value, HL test
(90)	C - R	China	West China Second University Hospital	Private/Mono.	179	Int.	-	—	—	77	AUC. KM, log-rank p-value, DCA
(107)	C - R	China	Fudan University Shanghai Cancer Center	Private/Mono.	117	N/A	N/A	N/A	N/A	N/A	AUC, Acc, C-index, KM, log-rank p-value
(91)	C - R	China	Fudan University Shanghai Cancer Center	Private/Mono.	160	Int.	-	—	—	57	AUC
(102)	C - R	China	Shengjing Hospital	Private/Mono.	182	Int.	—	-	—	79	C-index, NRI, log-rank p-value, KM, td-AUC, td-C-index
(94)	C - R	USA	TCIA ◦ TCGA	Public/Multi.	80	Ext.	China	Affiliated Huadu Hospital, China	Private/Mono.	37	C-index, KM, log-rank p-value
(89)	C - R	China	Anhui Provincial Cancer Hospital	Private/Mono.	101	Int.	-	-	-	40	AUC, Acc, Se, Sp, PPV, NPV, DCA, KM, log-rank p-value
(92)	C - R	China	Qilu Hospital of Shandong University	Private/Mono.	550	Int.	-	-	—	184	AUC, HR, KM, log-rank p-value, C-index
(93)	C - R	China	Hospital of Chongqing Medical University	Private/Mono.	92	Int.	-	-	-	56	AUC, Acc, C-index, KM, log-rank p-value, DeLong test
(25)	C - R	Korea	Samsung Medical Center	Private/Mono.	77	Int.	-	—	-	19	AUC
(98)	R - +	China	West China Second University Hospital	Private/Mono.	179	Int.	-	—	—	77	AUC, Se, Sp, DCA
(26)	H - O	USA	Fred Hutchinson Cancer Research Center ◦ Mayo ◦ University of Alabama at Birmingham	Private/Mono.	62 50 ◦ 46	Ext.	USA	TCGA	Public/Multi.	127	AUC, C-index, KM, log-rank p-value, HR, DeLong test

Center type — Mono., monocentric; Multi., multicentric. Modalities — R, Radiology; C, Clinical; H, Histology; O, Omics. Validation type — Int., internal; Ext., external. Cohort — N/A, no validation cohort reported; —, internal validation split; NR, Not Reported.

**Table 4:** Training and validation cohorts for multimodal studies (3 modalities).

TRAINING						VALIDATION					
Study	Modalities	Country	Dataset	Centers	Cohort	Type	Country	Dataset	Centers	Cohort	Metrics
(99)	C - R - O	USA	Memorial Sloan Kettering Cancer Center	Private/Mono.	108	N/A	N/A	N/A	N/A	N/A	OR, HR, log-rank p-value
(58)	O	USA	TCGA-OV	Public/Multi.	481 ◦ 292	Int.	–	–	–	192 ◦ 117	Acc, precision, recall, F1-score, KM, log-rank p-value, C-index, Brier score
(64)	C - R - O	UK	Addenbrooke’s Hospital	Private/Mono.	72	Int. ◦ Ext.	- ◦ UK	- ◦ Barts Health	- ◦ Private/Mono.	20 ◦ 42	RMSE, AUC, r_s, r_pb
(57)	C - R - H	China	Hospital A	Private/Mono.	111	Int. ◦ Ext.	- ◦ China	- ◦ Hospital B	- ◦ Private/Mono.	41 ◦ 31	AUC, Acc, Se, Sp, PPV, NPV, IDI, DeLong test
(27)	C - R - H	China	Yunnan Cancer Hospital	Private/Mono.	347	Int. ◦ Ext.	- ◦ China ◦ China ◦ China	- ◦ Hospital of Yunnan Province o Hospital of Chongqing Medical University o Zhongda	- ◦ Private/Mono.	87 ◦ 165 ◦ 90 ◦ 23	C-index, AUC, KM, log-rank p-value, HR

Center type — Mono., monocentric; Multi., multicentric. Modalities — R, Radiology; C, Clinical; H, Histology; O, Omics. Validation type — Int., internal; Ext., external. Cohort — N/A, no validation cohort reported; -, internal validation split; NR, Not Reported.

**Table 5:** Training and validation cohorts for multimodal studies (4 modalities).

TRAINING						VALIDATION					
Study	Modalities	Country	Dataset	Centers	Cohort	Type	Country	Dataset	Centers	Cohort	Metrics
(65)	C - O	USA	University of Iowa Hospitals and Clinics	Private/Mono.	88	Ext.	USA	TCGA HGSC	Public/Multi.	338	AUC, Se, NPV, HR
(48)	H - O	USA	TCIA ◦ TCGA ◦ TCPA	Public/Multi.	115	Int. o Ext.	China	- ◦ Tissue microarray	- ◦ Private/Mono.	114 ◦ 92	AUC, KM, log-rank p-value, HR
(24)	C - R - H - O	USA	Memorial Sloan Kettering Cancer Center ◦ TCGA	Private/Mono. ◦ Public/Multi.	404	Int.	–	–	–	40	C-index, KM, log-rank p-value, HR
(28)	C - R	China	Women’s Hospital, Zhejiang University	Private/Mono.	1742	Ext.	China	Hospital of Zhejiang University	Private/Mono.	102	AUC, Acc, Se, Sp, precision, F1-score, Brier score, Delong test

Center type — Mono., monocentric; Multi., multicentric. Modalities — R, Radiology; C, Clinical; H, Histology; O, Omics. Validation type — Int., internal; Ext., external. Cohort - N/A, no validation cohort reported; —, internal validation split; NR, Not Reported.

## 2 Multimodal data sources

Multimodal approaches leverage complementary data sources to capture tumor biology across multiple spatial and molecular scales. In HGSOC, these include image-based modalities such as radiological imaging and histopathology, as well as non-image-based data, including omics profiles (genomic, transcriptomic, and proteomic) and clinical variables. Each modality provides a distinct but partial view of the disease, reflecting different aspects of tumor burden, tissue architecture, and molecular mechanisms. This section provides a structured overview of the principal data modalities used in multimodal AI studies, before examining their respective contributions and the added value of their integration. These modalities correspond to the distinct data layers illustrated in **Figure 2**.

### 2.1 Imaging modalities

Imaging modalities capture macro-anatomical heterogeneity and play a central role in the clinical management of HGSOC. CT remains the cornerstone imaging modality for staging, treatment planning, and follow-up in advanced ovarian cancer. It provides a comprehensive overview of disease distribution, particularly for assessing peritoneal carcinomatosis (33). However, its diagnostic performance varies depending on lesion size and anatomical location (34). While CT is highly sensitive for detecting omental disease, it is less accurate for small peritoneal implants, subdiaphragmatic involvement, and bowel serosal or mesenteric disease (34). In addition, CT tends to underestimate overall tumor burden compared with intraoperative assessment, particularly in the presence of ascites or subcentimeter lesions. Its ability to detect lymph node metastases is also limited, as evaluation primarily relies on size-based criteria (34). These limitations may lead to underestimation of disease extent and suboptimal prediction of surgical resectability.

MRI, particularly when combined with diffusion-weighted imaging (DWI), has emerged as a valuable complementary modality for evaluating peritoneal disease (21). MRI offers superior soft tissue contrast and improved detection of lesions in anatomically challenging regions, including the upper abdomen, bowel serosa, and mesentery (35–38). Quantitative assessment of tumor burden using MRI-derived peritoneal cancer indices has shown strong correlation with surgical findings (39). As a result, MRI may improve preoperative assessment of disease extent and resectability, and has been included as the imaging gold standard to assess peritoneal carcinomatosis in ovarian and colon cancer (39).

PET/CT, provides functional information based on tumor metabolism and can enhance the detection of lymph nodes and extra-abdominal metastases. However, its sensitivity is reduced for small peritoneal lesions and for tumor subtypes with low metabolic activity (40, 41). Moreover, its added value in predicting surgical resectability at initial diagnosis remains limited, and it is not routinely recommended in standard clinical practice. Emerging approaches, such as PET/MRI and novel tracers targeting the tumor microenvironment (e.g., fibroblast activation protein) show promise but require further validation (42, 43). Overall, while imaging modalities are essential for clinical management, they primarily reflect macroscopic disease burden and have limited ability to capture the full biological complexity and spatial heterogeneity of HGSOC.

Given their inherently spatial nature, radiological images are rarely integrated directly into multimodal pipelines alongside tabular or molecular data. A common approach consists of projecting them into a scalar feature space prior to fusion. Radiomics, introduced and formalized in the context of personalized medicine by Lambin et al. (44, 45), are based on a set of quantitative descriptors from radiological images (CT, MRI, PET/CT) that characterize the textural, morphological, and statistical properties of tumor lesions. These extracted values enable a digital representation of the image’s textural richness, allowing for a more detailed and quantitative analysis than could be achieved with the human eye (further developed below).

### 2.2 Digital pathology

WSIs from digital pathology remain the diagnostic gold standard, providing a comprehensive view of tumor morphology and its microenvironment (26). In particular, hematoxylin and eosin (H&E)-stained slides are routinely used in clinical practice and play a central role in therapeutic decision-making, including chemotherapy initiation (24). WSIs capture rich histological information, such as tumor architecture, cellular morphology, proliferation patterns, and the distribution of tumor-infiltrating lymphocytes (46), while preserving alterations across both cellular and tissue scales (47, 48). Importantly, they retain the spatial organization of tissues, which is not accessible through genomic sequencing alone (24). In addition to H&E, complementary staining techniques such as immunohistochemistry (IHC) and fluorescence imaging provide deeper molecular insights by enabling the visualization of specific proteins and cellular markers but they are more expensive and less scalable for routine clinical use.

Recent advances in computational pathology have highlighted the potential of data-efficient and weakly supervised learning strategies to leverage WSIs at scale, enabling robust feature extraction without exhaustive annotations (49). Deep learning approaches have notably enabled the prediction of homologous recombination deficiency (HRD) and response to platinum-based therapy directly from histological slides in breast and ovarian cancers (50), highlighting the potential of WSIs as a non-invasive surrogate for molecular profiling. More broadly, pan-cancer computational histopathology has shown that histological patterns can reveal underlying mutations, tumor composition, and even patient prognosis (51). The emergence of foundation models as feature extractors for computational pathology further reinforces the scalability and generalizability of these approaches, particularly in weakly supervised settings (27, 52–55).

WSIs pose significant computational challenges due to their gigapixel-scale resolution, requiring patch-based processing strategies and substantial computational resources, often combined with filtering steps to reduce data volume (47, 48). Image quality may also be compromised by artifacts such as tissue folds, blurring, or staining impurities, necessitating manual quality control in some cases (26, 27). Furthermore, variability in scanners and acquisition protocols across institutions introduces potential sources of bias and limits model generalizability (26). To mitigate these effects, stain normalization techniques are commonly applied to harmonize color distributions across datasets, including widely used methods such as the Vahadane (56) and Macenko (57) algorithms.

Additional information such as nuclear-level features can be extracted from histopathology using automated segmentation. For example, Boehm et al. (24) use StarDist to generate tissue maps and derive morphological features such as tumor nuclear area, while Zeng et al. (48) apply CellProfiler to extract features related to nuclear shape and texture for survival prediction and molecular prediction.

### 2.3 Molecular profiling (omics)

Molecular profiling (omics) enables the characterization of tumor alterations across multiple scales: from single-cell to bulk tissue, and across different molecular layers, including DNA, RNA, and proteins, thereby improving our understanding of disease mechanisms (58). In the context of this review, each omics layer: genomic, transcriptomic, proteomic, epigenomic, is treated as an independent modality, in the same way that CT, MRI, and PET constitute distinct modalities within the radiological data. Numerous studies have leveraged these approaches to extensively characterize tumor biology (59–63).

Genomics characterizes the mutational landscape and chromosomal instability (48), while circulating tumor DNA (ctDNA) enables non-invasive monitoring of tumor burden and treatment response (64). Transcriptomics captures gene expression and regulatory programs through RNA sequencing data (65), whereas proteomics provides insights into the functional state of the tumor and its response to therapy (26). Epigenomics, through DNA methylation profiling and chromatin accessibility assays, reveals heritable regulatory alterations that modulate gene expression independently of sequence variation (65). Together, these approaches have contributed to define HGSOC by hallmark features such as the ubiquitous loss of TP53, extensive copy number alterations (61, 66–68) and a highly heterogeneous tumor microenvironment (TME) (7, 69, 70).

To date, genomic alterations support first-line treatment strategies primarily through the identification of BRCA1/BRCA2 mutations or, more broadly HRD, which confer increased sensitivity to PARP inhibitors (71, 72). Nevertheless, the definition of robust molecular subgroups in HGSOC remains a major challenge (60, 73–75), in sharp contrast to many other cancers where molecular subtypes routinely guide treatment decisions. Over the past decade, as omics technologies have matured and the investigation of fundamental cancer mechanisms has intensified, there has been a shift toward higher-resolution approaches. In particular, single-cell analyses have emerged as powerful tools to dissect tumor heterogeneity, revealing stromal dynamics and tumor-specific characteristics within the ovarian cancer microenvironment (69, 70, 76–79).

Recently, spatial transcriptomics (80, 81) has emerged as a promising bridge between imaging and molecular profiling, enabling gene expression to be mapped directly onto tissue architecture (7, 82–85). By providing spatially resolved molecular information, it addresses the lack of spatial context in traditional omics data and enhances the interpretability of approaches based solely on imaging. Compared to bulk omics approaches, single-cell technologies enable high-resolution dissection of tumor ecosystems by capturing cell–cell interactions and intra-tumoral heterogeneity (86). While bulk omics remain more cost-effective and scalable for biomarker discovery, they fail to resolve these fundamental cellular dynamics. Still, they remain high-dimensional, are often constrained by limited sample sizes, and still entail substantial acquisition costs (58). Despite their high scientific value, these technologies remain resource-intensive and are unlikely to be routinely implemented in clinical practice in the near future.

Nevertheless, large-scale spatial transcriptomics datasets generated in research settings constitute a valuable resource (87) for developing innovative computational approaches (88), particularly through multimodal integration, that could be incorporated into early clinical workflows.

### 2.4 Clinical and structured biological data

Clinical and structured data are widely used to build predictive models of survival and treatment response. Key variables include, among others, the FIGO stage, e.g. (57, 89, 90), serum biomarkers such as CA-125, e.g. (91–93), and patient characteristics such as age at diagnosis, e.g. (91, 94). Tumor-related features, including size, laterality, extent of peritoneal disease, and residual tumor status after surgery, e.g. R0 resection, are also established prognostic factors and are commonly incorporated into predictive models, e.g. (27, 57, 65, 90, 92, 93, 95, 96).

Similarly, clinical indicators such as platinum-free interval and performance status (e.g., ECOG) are frequently used to stratify patients and guide therapeutic decisions. Molecularly informed clinical variables, including BRCA mutation and HRD status, further refine prognostic assessment and treatment selection; they are now integrated into routine diagnostic and therapeutic workflows.

In addition to static variables, dynamic biomarkers have emerged as important predictors of treatment response. Among these, the CA-125 elimination rate constant (KELIM) has been shown to robustly reflect tumor chemosensitivity and is associated with survival outcomes and response to therapies such as platinum-based chemotherapy and PARP inhibitors (97). KELIM, derived from longitudinal CA-125 kinetics during the first 100 days of platinum-based chemotherapy, has been repeatedly validated as an early, reproducible indicator of intrinsic chemosensitivity, independently predicting radiologic response, feasibility of complete interval debulking, risk of platinum-resistant relapse, progression-free survival, and overall survival across large trial datasets and real-world cohorts (97).

Some pipelines also incorporate expert-defined scores. Chen et al. (98), propose a lymph node score based on CT scan (*CT LN report*), in which radiologists translate image interpretation into a binary variable that can be combined with other information. Moreover, these variables rely on subjective assessments, leading to inter-observer variability (99).

These clinical and biological data are routinely collected in clinical practice, facilitating their integration into predictive tools such as nomograms and machine learning models. However, they are often incomplete or inconsistently reported, particularly in retrospective cohorts, and remain insufficient to capture the full biological complexity and heterogeneity of HGSOC when used in isolation (65).

### 2.5 The value of multimodal integration

The integration of multiple modalities has become an essential approach for a deeper understanding of intricate diseases like HGSOC, utilizing diverse information across various biological levels.

First, multimodal data provide different types of information at various biological levels. Each type focuses on specific aspects of the disease, and combining them gives a global representation (24). Radiological modalities, such as MRI and CT, capture the global tumor burden, spatial heterogeneity, and organ invasion, while histopathology provides insights into tissue architecture and the tumor microenvironment. At a finer level, omic data reveal the biological mechanisms. Clinical variables further complement these modalities by reflecting the patient’s overall health condition (27).

Additionally, multimodal integration is crucial for understanding tumor heterogeneity, a characteristic of HGSOC marked by both genetic and morphological variability (100). Recent studies suggest that combining multimodal data could effectively address tumor heterogeneity from various dimensions (101). For instance, Nougaret et al. (99) demonstrate a correlation between CT imaging characteristics and BRCA mutation status, indicating that tumor heterogeneity exists at both molecular and macroscopic levels. Moreover, they reveal that imaging phenotypes, genomic alterations, and clinical outcomes are closely linked, highlighting the complementarity of these modalities.

Then, predictive capabilities can be increased with the implementation of multimodal integration (101). Indeed, most studies consistently show that multimodal models outperform unimodal approaches (25, 64, 65, 102). For example, the use of MRI with histopathology images enhances response assessment (57); while integrating CT radiomics, clinical, and genomic (ctDNA) data yield improved predictions of global tumor volumetric change during NACT in patients with HGSOC (64). Histopathology appears to be an especially useful modality when combined with one or more of the various types of data (e.g. imaging, proteomic or genomic), and consistently improves the prediction of overall survival, progression-free survival, and platinum sensitivity (24, 26, 27, 48, 57). Indeed, Kilim et al. (26) reveal through their three distinct fusion paradigms, that histology and proteomics are highly synergistic, and their aggregation can outperform the HRD score to predict response and survival. Moreover, the use of combinations of different types of imaging provides complementary diagnostic results, thus improving clinical accuracy (28).

Finally, multimodal approaches improve robustness when data are incomplete, which is crucial in real-world clinical settings (27). Additionally, they enhance interpretability by finding morphological patterns that showcase molecular processes, and providing more biologically and clinically interpretable predictions (26, 99).

Overall, these observations highlight that multimodal integration is not only beneficial but essential for capturing the multi-scale nature of tumor heterogeneity and improving decision-making in oncology. However, assembling large cohorts with all modalities remains challenging, and most studies face the “curse of dimensionality”, in other words more features than patients. Despite this limitation, establishing robust methodological foundations now is essential to leverage future large-scale multimodal datasets as they become available through prospective multicentric initiatives or federated learning.

## 3 Modality-specific processing pipelines

This section describes how each modality is processed from raw data into a patient-level representation through key stages including preprocessing, feature extraction, and intra-modal aggregation; the two latter stages being represented respectively by color and shape in **Figure 2**.

### 3.1 Per-modality preprocessing

Before any feature extraction is performed, the raw data from each modality go through specific preprocessing steps for each modality, aimed at standardizing the input data.

#### 3.1.1 Radiology

Tumor ROI segmentation is the central and most labor-intensive preprocessing step across all radiological studies, performed manually in the vast majority of cases (24, 25, 27, 57, 89–93, 95, 96, 98, 103–107) and only semi-automatically in a few (28, 64, 94, 102, 108). Voxel resampling to isotropic resolution and intensity normalization are standard in MRI-based studies (25, 27, 57, 89, 91, 107), while CT-based studies additionally apply HU windowing and intensity discretization before radiomic extraction (24, 64, 103, 104). A notably advanced preprocessing is found in (57), which performs habitat imaging, i.e. voxel-wise k-means clustering across multiple MRI sequences to delineate intra-tumoral sub-regions, in order to capture spatial heterogeneity beyond standard ROI-level analysis. PET-based preprocessing, represented by a single study (102), follows a similar pattern to CT, with maximum standardized uptake value (SUVmax)-based thresholding for tumor VOI definition and fixed-bin intensity discretization prior to radiomic extraction. US used exclusively in (28), relies on manual ROI segmentation via ITK-SNAP, with no additional preprocessing reported.

#### 3.1.2 Histology

WSI preprocessing follows a largely standardized pipeline across studies: tissue segmentation or background removal, patch extraction at 20× magnification, and patch filtering based on tissue content thresholds (24, 26, 27, 47, 48, 56, 57, 100). Stain normalization (Macenko or Vahadane) is applied in several studies to reduce inter-center staining variability (24, 56), though notably absent in foundation model-based approaches such as (26), where pre-trained encoders are assumed robust to staining variation. Manual quality control by pathologists remains common (26, 27, 47, 57, 100, 109), reflecting the persistent need for expert curation despite automated pipelines. A particularly original step is found in (24), which combines Convolutional Neural Networks-based (CNN) tissue classification with nuclei segmentation to generate cell-type-level features, going beyond standard patch-level representations.

#### 3.1.3 Omics

Preprocessing strategies vary substantially depending on the omics type. Genomic data is typically encoded as binary mutation vectors after variant calling (24, 48). Transcriptomic and proteomic data require distinct normalization strategies, encompassing a broad spectrum of methods ranging from simple scaling procedures to advanced model-based and variance-stabilizing techniques. Furthermore, embedding-based dimensionality reduction of these high-dimensional matrices provides an efficient framework for their integration into multimodal models, enabling compact and informative representations (26, 48). The most rigorous omics preprocessing pipeline is found in (65), which integrates single nucleotide variation (SNV) calling, mRNA expression, methylation analysis, and fusion gene detection across four omics layers.

#### 3.1.4 Clinical data

Clinical variables require minimal preprocessing. Typically direct use as raw tabular features, with occasional z-score normalization (93, 107), median imputation for missing values (28, 89), and categorical encoding (24, 64, 96, 107). Several studies report no explicit preprocessing of clinical variables (25, 95, 99, 105).

A recurring pattern across modalities is the coexistence of automated pipelines with mandatory manual steps, particularly tumor segmentation in radiology and slide selection in histopathology.

This dependence on expert annotation represents a shared bottleneck limiting scalability and reproducibility across studies.

### 3.2 Feature extraction

Once preprocessed, each modality is transformed into a concise and informative representation using modality-specific feature extraction strategies, from manually defined radiomic descriptors to deep learning-based (DL) encoders.

#### 3.2.1 Radiology

Radiomics analysis, the process of converting digital medical images into mineable high-dimensional data, is motivated by the concept that biomedical images contain information reflecting underlying pathophysiology that can be revealed through quantitative image analysis (89). Radiomic feature extraction is the dominant approach across CT, MRI, PET, and US studies (24, 25, 28, 57, 64, 90, 91, 94–96, 98, 102, 104, 107, 108), yielding shape, intensity, and texture descriptors (106) including gray-level co-occurrence matrix (GLCM) and wavelet-based features (103). Manual or expert-driven extraction remains present in early studies (99, 106). A feature selection pipeline, often combining redundancy filtering (Spearman correlation), reproducibility filtering, intra- or interclass correlation coefficients (ICC), and LASSO regularization, is applied in most radiomics-based studies (24, 25, 28, 57, 89–91, 94, 96, 98, 102, 104, 107, 108), typically reducing hundreds of initial features to a compact predictive signature. DL-based extraction is used in a minority of studies: Wang et al. (105) employs a CNN autoencoder producing 16-dimensional latent vectors, and Zheng et al. (92) leverages an ImageNet-pretrained vision transformer (ViT) generating 128-dimensional semantic representations. Wei et al. (96) enriches radiomic extraction by computing features on both the original CT and eight wavelet decompositions, substantially expanding the feature space. An interesting approach is proposed in (57), which applies habitat imaging, extracting radiomics from k-means-defined intra-tumoral sub-regions, capturing spatial intra-tumoral heterogeneity at a finer scale than standard whole-ROI radiomic analysis, where all voxels are treated as a single homogeneous region. Furthermore, Bi et al. (27) leverages the pre-trained radiological foundation model MedSAM (110) for MRI feature extraction, combining the robustness of large-scale pre-training with the specificity of medical imaging, a paradigm shift that reduces dependency on large annotated cohorts.

#### 3.2.2 Histology

Feature extraction from WSIs is dominated by DL encoders applied to patches. CNN-based encoders are used in earlier studies (56, 100, 109), while more recent works adopt ViT-based foundation models pre-trained on large pathology datasets, such as UNI (26) or CONCH (27), significantly reducing the need for large task-specific training cohorts, a critical advantage given the limited cohort sizes typical of ovarian cancer studies. Unlike CNNs, ViT-based encoders rely exclusively on attention mechanisms to establish global dependencies across the entire image, enabling simultaneous consideration of all patch regions and potentially better capturing complex spatial relationships (57). Self-supervised contrastive learning (SimCLR) is used in (47) as an alternative to supervised pre-training, leveraging unlabeled data to learn discriminative patch representations. In contrast, method introduced in (48) relies on handcrafted morphological and texture features extracted via CellProfiler, including Haralick, Gabor, and Zernike descriptors. Importantly, histopathological feature extraction in (24, 48) goes beyond standard radiomic descriptors by capturing cell-level and microenvironmental information, such as nuclear morphology, cell density, and tissue composition, a level of biological granularity inaccessible to radiological imaging, which characterizes tumor anatomy at a macroscopic scale.

#### 3.2.3 Omics

Feature extraction strategies vary considerably across omics types. For genomic data, approaches range from direct use of raw mutation status (99) and binary mutation encoding (24) to selection of the 100 most frequent somatic mutations as a compact feature vector (48), while ctDNA-based studies derive quantitative instability metrics such as Minor Allele Frequency (MAF) and t-MAD from targeted sequencing (64). Transcriptomic data is processed through normalization and differential expression analysis, commonly using the DESeq2 package (111), to select the most informative genes (48, 65). Proteomic features are used more directly, either as raw reverse phase protein array (RPPA) analysis (48) or derived from a curated biological signature called the Chowdhury signature, after cross-cohort intersection and normalization (26). The most sophisticated omics extraction pipeline is found in (58), which trains a variational autoencoder to produce compact latent representations from genomic, transcriptomic, and epigenomic data jointly. In contrast (65), applies a purely statistical selection pipeline: ANOVA followed by LASSO, across four omics layers simultaneously.

#### 3.2.4 Clinical data

Clinical variables occupy a unique position in the pipeline: unlike imaging or omics data, they are already patient-level representations, making preprocessing and feature extraction inherently coupled. Consequently, most studies use clinical variables directly as raw tabular features (25, 28, 64, 89, 91, 92, 94–96, 99, 107), with the preprocessing steps such as encoding, normalization, imputation, effectively constituting the extraction step itself. Some authors propose a feature selection step (24, 57, 65, 90, 102, 105). Only a small number of studies apply a dedicated learning-based extraction module on top of clinical variables: Liu et al. (93) projects them through a fully connected layer into a learned embedding, while Bi et al. (27) encodes them via a multilayer perceptron (MLP) after Cox-based variable selection. A shared pattern across modalities is the application of feature selection pipelines such as LASSO, Cox univariate filtering, or correlation-based pruning, to reduce high-dimensional feature spaces before fusion, particularly valuable given the small patient cohorts inherent to HGSOC studies.

Deep learning and foundation model-based extractors implicitly perform this dimensionality reduction through learned representations, whereas radiomics-based pipelines require explicit selection steps.

### 3.3 Intra-modal aggregation mechanisms

A crucial but rarely addressed step in multimodal pipelines is the aggregation of modality-specific features into a single representation at the patient level. Unlike clinical or omics data, which are often inherently patient-level, imaging data such as WSI images or multi-lesion CT/MRI scans require an explicit aggregation step to produce a unified representation per modality and per patient. This intermediate representation is not a final prediction, but a compact signature that will subsequently be combined with other modalities. The aggregation strategy chosen at this stage directly determines the quality and relevance of the downstream fusion.

#### 3.3.1 Radiology

For radiological modalities, intra-modal aggregation is required when multiple lesions or multiple image slices are present within a single patient. In several studies, a single lesion or ROI is defined per patient, making aggregation unnecessary because features are directly used as patient-level representations (28, 91, 95, 99, 104). When aggregation is needed, the most common approach is deterministic aggregation: unweighted averaging across lesions (64, 105) or across image slices (93). A more informative variant is volume-weighted averaging, used in (25), where lesion contributions are weighted proportionally to their volume: a clinically motivated choice that prioritizes larger, potentially more aggressive lesions. In the specific context of radiomic pipelines, intra-modal aggregation is often implicit in the construction of a Rad-score: a weighted linear combination of selected radiomic features, where coefficients are learned through machine learning (ML) methods such as logistic regression or LASSO, producing a single uninterpretable scalar per patient (89, 90, 94, 96, 98, 102, 107, 108). Similarly, Boehm et al. (24) fits a univariate Cox proportional hazards model on CT radiomic features to derive an interpretable risk score per patient. A notable exception is approaches proposed in (106) and (103), which construct an inter-site similarity matrix (ISM) by computing pairwise Euclidean distances between lesion-level feature vectors, then derive a compact set of heterogeneity metrics, capturing inter-lesion texture variability rather than averaging it away. Zheng et al. (92) stands out by using a recurrent neural network (RNN) to sequentially integrate slice-level feature representations into a patient-level risk score, explicitly modeling the spatial ordering of CT slices. In addition, Bi et al. (57) applies a Random Forest on habitat-level radiomic features to construct a patient-level habitat signature, while they applies a self-attention network to compute a weighted average of slice-level MRI feature vectors, mirroring its attention-based aggregation used for WSIs in (27).

#### 3.3.2 Histology

Aggregating patch-level features into a single slide- or patient-level representation is the central challenge of WSI-based pipelines. Multiple Instance Learning (MIL) has emerged as the dominant framework for this task, treating the WSI as a bag of patch instances and learning to identify prognostically relevant regions that may be missed during subjective pathologist review (47). Attention-based MIL variants, which weight each patch by a learned attention score, are increasingly adopted in HGSOC studies (26, 56). Bourgade et al. (56) computes the slide representation as a weighted sum of patch feature vectors using attention scores, while Kilim et al. (26) employs CLAM’s attention-based pooling function to aggregate patch-level features into slide level representations. A more complex aggregation is found in (47), which constructs multi-scale feature pyramids by concatenating embeddings across different magnification levels before MIL aggregation. In contrast, Bi et al. (27) uses a self-attention network with learnable parameters to compute weighted averages of patch and slice features, enabling explicit and interpretable modality level weighting. In another approach (57), Bi et al. combine Patch Likelihood Histogram and Bag-of-Words representations to summarize the distribution of patch-level predictions, before applying a Random Forest classifier to derive the final WSI-level pathology signature. Boehm et al. (24) goes beyond patch-level aggregation by integrating both tissue-level and cell-level features into a unified histological risk score, capturing biological information at multiple scales with a Cox proportional hazards model. In addition, a simpler deterministic strategy can be adopted, such as averaging features across 60 randomly sampled patches, which, while computationally efficient, does not discriminate between informative and non-informative regions (48). Finally, an implicit aggregation through the final layers of their CNN architectures, without any explicit pooling mechanism is proposed in (100, 109).

#### 3.3.3 Omics and clinical data

For omics and clinical variables, intra-modal aggregation is generally not required, as features are already defined at the patient level. These modalities therefore proceed directly to the inter-modal integration stage (48, 58, 64, 65, 99). For proteomic data, Kilim et al. (26) applies a dense neural network to project the selected protein features into a compact learned representation, rather than using them directly. For clinical and genomic data, Boehm et al. (24) derives modality-specific risk scores via Cox proportional hazards models, except for genomic data, where HRD/HRP status is directly binary-encoded. To ensure a comparable scalar representation for downstream fusion, Li et al. (107) similarly produces a non-interpretable clinical risk score via support vector machines.

## 4 Multimodal fusion frameworks

Multimodal fusion frameworks define how heterogeneous data sources are combined into a predictive model. This section first presents fusion strategies across the various stages of the pipeline, followed by inter-modal integration and prediction mechanisms that determine how information is combined across different modalities. All technical details are summarized and visually illustrated in **Figure 2**.

### 4.1 Fusion strategies across the pipeline

The main advantage of multimodal models is that distinct modalities can provide complementary information and they can be integrated at different stages of the processing pipeline. As shown in **Figure 3**, there are three main types of fusion strategies, which differ from one another based on the stage at which the modalities are combined. The fusion strategies selected in each article in this review are represented in **Figure 2** by the style of the lines. The following are descriptions of the three most common fusion strategies:

#### 4.1.1 Early fusion

This fusion combines data from different modalities *M_i_* directly, prior to any learning process, into a single input which is then processed by either a trainable model or a direct predictive function. In this review, we propose to distinguish two subtypes based on the nature of the data being merged. *Raw tabular-level fusion* concatenates raw modality data directly, without any prior projection or transformation (58):


$$M=\left[M_{1}|M_{2}\left|\ldots \right|M_{n}\right]$$

This approach is only applicable when all modalities are natively tabular, such as clinical variables or certain omics profiles, since their scalar values share a common numerical structure that allows easy vector concatenation.

Strengths: simplest to implement; allows the model to learn cross-modal correlations directly from raw features; no information loss due to feature extraction.

Limitations: limited to natively tabular modalities; sensitive to scaling differences across modalities; requires complete data for all modalities; limited interpretability.

In practice, this strategy cannot generalize to all medical data, given the inherent heterogeneity of clinical modalities; imaging data in particular cannot be trivially combined with tabular inputs in its raw form. *Feature-level fusion* addresses this limitation by first extracting modality-specific scalar features *F_i_*, such as radiomic features or genomic variables, through dedicated encoders or pipelines. The resulting interpretable representations share the same scalar nature and can then be meaningfully concatenated into a unified input vector (25, 28, 48, 64, 65, 91, 95, 99). This makes feature-level fusion the dominant early fusion strategy in multimodal medical AI, as it accommodates the inherent heterogeneity of clinical data while preserving the simplicity of a single joint input.


$$F=\left[F_{1}|F_{2}\left|\ldots \right|F_{m}\right]$$

Strengths: applicable to heterogeneous modalities; encoder-derived representations can provide richer, task-agnostic information than hand-crafted features; preserves the simplicity of a single joint input.

Limitations: sensitive to scaling differences; requires temporal and spatial alignment between modalities; limited interpretability and requires complete dataset.

#### 4.1.2 Intermediate fusion (representation-level fusion)

This strategy integrates the intermediate representations or modality-specific embedding *Z_i_* obtained by applying a feature extractor *E_i_* to each input *M_i_*, such that:


$$Z_{i}=E_{i}\left(M_{i}\right)$$

These representations are then combined and used as inputs to a trainable model or a final predictive model. In this case, *Z_i_* corresponds to latent embeddings that are not directly interpretable and cannot be directly used as final predictions. This strategy is increasingly adopted in recent work, e.g. in (26, 27, 57, 93, 107), particularly those employing attention mechanisms, reflecting the shift toward learned cross-modal representations. For example, the CHP framework, proposed in (57), utilizes multi-head attention to dynamically weight modality based on their relevance to a specific patient’s prognosis. A common and specific instantiation of this strategy is the radiomics score (Radscore), a weighted linear combination of selected radiomic features (89, 90, 94, 96, 98, 102). Despite being derived from interpretable handcrafted features, the Rad-score constitutes a compressed latent representation because it requires learning its coefficients, so it is unsuitable for direct clinical interpretation, placing it within the intermediate fusion paradigm.

Strengths: Capable of modeling complex inter-modal interactions while reducing dimensionality and handling heterogeneous high-dimensional data.

Limitations: Loss of interpretability; final predictions depend on learned latent representations; requires significant computational resources and appropriate regularization to avoid overfitting.

#### 4.1.3 Late fusion (decision-level fusion)

This fusion combines the final predictions *Y_i_* produced independently by modality-specific models using an aggregation function *P* such as weighted combinations or cox model (24, 92, 105), resulting in a final prediction score *Y*:


$$Y=P\left(Y_{1},Y_{2},\ldots ,Y_{n}\right)$$

In contrast to intermediate fusion, each modality corresponds to complete predictions that could be used independently, and the inter-modal integration stage acts as an ensemble method.

Strengths: Robust to missing data as each modality can contribute independently to the final decision, and generally offers better interpretability.

Limitations: May fail to capture subtle correlation between modalities that only occur at earlier stages and can suffer from dominance of a single modality in the final prediction.

### 4.2 Inter-modal integration mechanisms and prediction

Regardless of the chosen fusion strategy, once intra-modality aggregation is performed (when required), patient-level representations from each modality must be integrated. This stage, referred to as inter-modal integration, combines patient-level modality-specific embeddings into a unified representation. **Figure 2** presents the proposed taxonomy of inter-modal integration, illustrated using color-coded lines. At the end of each unimodal or multimodal pipeline, a Prediction Head is applied to produce the final output according to the targeted clinical task. In **Figure 2**, the prediction head category is encoded by the symbol positioned at the rightmost end of each line, which can be a cross (×), or one, two, or three left chevrons (<, ≪, <<<).

The six categories of Inter-modal Integration taxonomy are described below:

- Concatenation-based fusion consists of directly concatenating patient-level representations from each modality into a single vector, without any learned interaction between modalities (58, 64, 93). While straightforward to implement, this approach does not explicitly model cross-modal dependencies.
- Heuristic fusion relies on mathematical, non-learned integration rules to combine patient-level representations, such as weighted averaging with fixed coefficients (107).
- ML-based integration combines modality-specific representations through a statistical or ML model, most commonly multivariate Cox proportional hazards regression (24, 92, 94, 96, 102, 105), or multivariate logistic regression (89, 91, 95, 99). All of these fusion approaches implicitly involve implicit weighting where feature importance is learned from the data. In these cases, the integration and prediction steps are tightly coupled, as the same model simultaneously fuses modalities and produces the final output.
- Attention-based integration dynamically weights embeddings across modalities to identify the most informative modality for each patient, enabling adaptive and patient-specific integration. This can be implemented through weighted sum using attention score such as (27), or with multi-head cross-attention module (57). Indeed, since different modalities describe the disease at different scales, conventional integration methods often fail to fully leverage their complementarity. Multi-head attention (57) addresses this limitation by integrating multi-level features while adapting their relative importance.
- Tensor-based fusion, exemplified by the Kronecker product used in the method proposed in (26), explicitly models multiplicative interactions between modality-specific representations, capturing higher-order cross-modal correlations that concatenation-based methods cannot express.
- Not Reported: a subset of studies does not explicitly describe their inter-modal integration mechanism (25, 28, 48, 90, 98), limiting reproducibility and methodological comparison across studies.

Once inter-modal integration is complete, the resulting unified patient-level Multimodal representation is passed to a prediction head, which maps it to the targeted clinical output. The four types of Prediction Head are described below:

- ML-based predictors are the most widely used in HGSOC studies, reflecting the dominance of survival analysis and treatment response tasks in this field. Several studies apply clustering or stratification methods to derive patient risk groups, including k-means (106, 108) and median dichotomization (103). Regression-based predictors are the most prevalent, ranging from elastic net logistic regression (104) and multivariate LASSO regression (65) to unweighted ensemble regression (64) and Cox-based statistical models (58, 99). Tree and ensemble methods are also represented, including XGBoost (25), random forest (48), and voting-based strategies (47). Finally, some studies report the use of a ML model without further specification (28).
- DL-based predictors leverage neural network architectures to map the unified multimodal representation to the final clinical output. Several studies employ a final linear or fully connected layer applied directly to the patient-level representation (56, 57), while others use a dedicated multilayer perceptron (27) or a more complex neural network (26). CNN-based predictors are also used, either as a standalone regression output (109) or as a secondary CNN applied to the aggregated representation (93, 100).
- Included in Inter-modal Integration: in many studies the prediction step is not separable from the inter-modal integration, as the fusion mechanism simultaneously produces the final output, particularly when based on ML or heuristic methods, as described below.
- Not Reported: a small number of studies do not explicitly describe their prediction mechanism (90, 98).

## 5 Explainability in multimodal AI models

Deep learning models, particularly in multimodal settings, are still challenging to interpret, which constitutes a major barrier to clinical use (112). The “black-box” nature of these types of models limits the trust by clinicians, who need to have transparent and auditable decision-support tools. In HGSOC, where treatment decisions have a direct impact on survival, toxicity, and quality of life, interpretability is essential to ensure safety and clinical acceptance. Consequently, explainability methods such as Grad-CAM (109) or SHapley Additive exPlanations (SHAP), have become increasingly important to verify that models rely on clinically relevant features (32, 113).

### 5.1 Radiology

In radiology, interpretability is often achieved with feature-based methods. SHAP values can quantify the contribution of radiomic and clinical features in models such as XGBoost, providing insight into prediction drivers (25). In addition, nomograms combine radiomic scores with clinical variables to produce visual and clinically interpretable tools to aid in clinical decision-making (90, 91, 94, 96, 98).

### 5.2 Histology

Several approaches provide visual interpretability. Grad-CAM highlights regions contributing to final predictions, often corresponding to tumor cell clusters and their organization (109). In the model presented by Laury et al. (100), the explainability is ensured by the identification of digital biomarkers that are linked to the tumor regions in response to the treatment. These regions are then reviewed and validated by a pathologist, ensuring that the highlighted patterns are morphologically meaningful and clinically interpretable. In addition, attention-based mechanisms can also highlight biologically meaningful regions: while Kilim et al. (26) uses attention maps to link proteomic signals to specific areas within a multimodal co-attention framework validated by pathologists, Ahn et al. (47) relies on the attention mechanism within a MIL framework to extract and visualize high-attention patches associated with platinum response.

### 5.3 Omics

Interpretability is typically addressed by linking latent features back to biological variables. This includes correlation-based mapping to genomic and transcriptomic features (58) and functional interpretation through Gene Ontology enrichment analysis (48).

Finally, in multimodal models, explainability can be achieved by analyzing the feature importance within ensemble frameworks, and identifying key contributors such as treatment variables or radiomic features related to tumor heterogeneity (64).

## 6 Current challenges and discussions

### 6.1 From unimodal radiomics to true multimodal integration

Prior to the radiomics era, prognostic scores were derived manually by radiologists through subjective qualitative assessment (99). The formalization of radiomics (45) triggered a wave of CT and MRI-based studies, explaining the dominance of two-modality approaches from 2017 onwards in **Figure 2**. Studies incorporating omics data tend to be published later (after 2021; cf. **Figure 2**), reflecting the greater complexity of acquiring such data (58, 65) and integrating them with heterogeneous data. This is a challenge further exacerbated in the case of HGSOC, where omics datasets are scarce. The standard strategy is to pool data from different types of cancer (58).

### 6.2 Foundation models: promises and limitations

Despite the promising results achieved by foundation models such as CONCH, MedSAM, and UNI in the studies reviewed (26, 27), and considering the broader foundation model literature across cancer types, several critical limitations must be acknowledged before these approaches can be considered ready for clinical deployment. Their substantial computational requirements restrict accessibility in low-resource settings (114), while their black-box nature complicates regulatory approval under frameworks mandating explainability for high-risk medical AI (114). Hallucinations represent a particularly serious concern in oncology: foundation models can generate factually incorrect outputs that may propagate into flawed prognostic conclusions and erode clinician trust (115). Domain shift further limits generalizability, as models pre-trained on pan-cancer datasets do not necessarily transfer to HGSOC-specific patterns (116, 117); indeed, Kilim et al. (26) showed that an ovarian cancer-specific self-supervised model matched UNI on TCGA-OV. Critically, the assumed superiority of foundation model features over conventional alternatives is not universal: Demircioglu (118) found no significant performance advantage over hand-crafted radiomic features across ten radiological datasets. Rigorous prospective validation and interpretability frameworks remain prerequisites for clinical translation.

### 6.3 What counts as multimodal?

Several ambiguities recur across reviewed studies. First, several papers classified as unimodal integrate multiple sequences of the same modality such as T1W, T2W, DWI, ADC (25, 27, 28, 89, 107), blurring the boundary between unimodal and multimodal. Second, most two-modality studies combine imaging with clinical variables, for example (25, 94, 95, 102), which augments traditional clinical risk models rather than performing true cross-modal integration. Additionally, several papers include omics or transcriptomic data solely for biological interpretation without integrating them as model inputs (47, 103, 106, 109), complicating cross-study comparisons. Beyond the number of modalities, the depth of integration also varies, particularly for genomic data, ranging from a single binary feature (24) to hundreds of jointly encoded multi-omics variables (58). Such methodologically disparate approaches, differing in complexity, should not be treated as equivalent in comparative analyses, as a single genomic feature carries substantially different information content than a comprehensive multi-omics signature.

### 6.4 Reproducibility and methodological transparency

Several clinically-oriented papers describe inter-modal integration vaguely as a “combined model” without specifying the integration mechanism (90, 98), precluding reproducibility. While code availability has improved in recent years (24, 26–28, 47, 57, 58, 64, 105, 109), data sharing remains rare due to privacy constraints, making independent replication virtually impossible even when code is provided. In parallel, the heterogeneity of performance metrics reported across reviewed studies, spanning non-comparable cohorts and validation protocols, precludes any formal quantitative synthesis (see **Tables 2**–**5**). This methodological fragmentation constitutes an important finding of this review, highlighting the urgent need for standardized evaluation frameworks and shared benchmarks in AI-driven ovarian cancer research. Beyond these transparency and reproducibility issues, none of the 31 included studies explicitly reported adherence to formal quality assessment frameworks such as PROBAST, TRIPOD-AI, CLAIM, or the Radiomics Quality Score, precluding a reliable and standardized comparative quality appraisal across the corpus and further reflecting the broader absence of reporting standardization in this emerging field.

### 6.5 Validation practices and the TCGA illusion

Validation practices, summarized in **Tables 2**–**5**, vary widely across reviewed studies: 6 out of 31 report both internal and external validation (27, 48, 57, 64, 96, 105), 12 rely solely on internal validation (24, 25, 58, 89–93, 98, 100, 102, 109), 8 on external validation only (26, 28, 47, 56, 65, 94, 104, 108), and 5 provide no validation at all (95, 99, 103, 106, 107), though the latter partially mitigate this through leave-one-out cross validation (106, 107). The widespread use of TCGA and TCIA (26, 47, 48, 56, 65, 94, 108, 109) raises additional concerns regarding true generalizability. Although studies frequently refer to held-out TCGA subsets as “external validation”, these datasets often share similar acquisition protocols, preprocessing pipelines, demographic composition, annotation practices, and institutional biases. As such, they should be considered closer to internal or cross-institutional validation rather than true external validation in the clinical sense. Importantly, TCGA ovarian cancer cohorts also predominantly reflect retrospective datasets acquired more than a decade ago, limiting their representativeness with respect to contemporary imaging protocols, molecular assays, and current therapeutic strategies. Another major limitation is the underrepresentation of geographic, ethnic, and socioeconomic diversity across available cohorts. Most multimodal studies remain heavily biased toward North American and European populations, raising concerns regarding demographic imbalance and reduced applicability to broader global populations. Finally, few studies evaluate model calibration or robustness under real-world deployment conditions, despite calibration being essential for clinical decision-making. The absence of standardized multicentric benchmarks with harmonized reporting metrics remains a major obstacle for objective comparison and clinical translation of multimodal AI models in HGSOC. Future efforts should prioritize prospective multicentric cohorts, geographically diverse populations, transparent reporting standards, and federated validation frameworks specifically designed to evaluate robustness across institutions and healthcare systems.

### 6.6 Missing modality robustness

Most studies simply exclude patients with incomplete data, further reducing already limited sample sizes. Only Bi et al. (27) treats missing modality robustness as a central contribution, validating performance under incomplete modality scenarios through crossmodal attention. A few studies apply explicit imputation (26, 28, 64, 89), while Boehm et al. (24) adopts late fusion as an implicit robustness strategy. The vast majority offer no dedicated approach. These strategies reflect the current state of the HGSOC literature, but missing data is a challenge shared across oncology. Broader research has proposed complementary solutions: transfer learning from large external datasets across various cancer types including colon (119), breast (120, 121), and bladder cancer (122), self-supervised pretraining on unlabeled data (123–126), text embedding integration for knowledge transfer in low-data regimes (54), federated and swarm learning for privacy-constrained multi-center settings (127, 128), and synthetic data augmentation via generative models (129). Systematically adopting such strategies in future HGSOC studies could substantially improve model robustness under real-world clinical conditions.

### 6.7 Interpretability as a prerequisite for clinical adoption

Model interpretability is increasingly recognized as a prerequisite for clinical trust, yet explicit explainable analysis remains rare. Only four studies employ named techniques: Grad-CAM (109), SHAP (25), and attention maps (26, 47), fewer than 15% of reviewed papers. Beyond HGSOC, explainability has emerged as a critical and actively debated challenge across oncology. In computational pathology, Layer-wise Relevance Propagation and attention-based heatmaps have been used to localize morphologically relevant tissue regions for biomarker predictions (130). Foundation model benchmarking has further demonstrated that different models attend to distinct tissue areas despite reaching similar predictions, underscoring the need for systematic explainability evaluation rather than performance-only assessment (131). In the broader ovarian cancer ML literature, SHAP has been applied to identify clinically key predictors (132–134), while LIME has been proposed as a complementary approach to improve transparency in ensemble classifiers (135). More recent reviews position XAI (32), encompassing SHAP, Grad-CAM, LIME, and GNN Explainer, as a regulatory prerequisite for clinical deployment, noting that without explainability, AI models risk perpetuating hidden biases and cannot meet FDA and EMA transparency requirements (112, 136). This gap reflects both the technical complexity of multimodal explainability and the resource constraints of academic research groups.

### 6.8 State of multimodal analysis in other cancer types

The multimodal pipeline taxonomy introduced in this review is not specific to HGSOC and extends to other cancers. Studies such as (137–139) extract multi-scale histological and MRI features in breast and colon cancer. Although rarely detailed, intra-modal aggregation is explicitly described in (27) and (140), the latter summarizing histopathology at slide level and gene expression at pathway level, while the former uses attention-based weighting. Inter-modal integration mainly relies on either ML-based methods (e.g., Cox regression or support-vector-machine in (141, 142)) or Attention-based approaches (138, 143), including more recent Key–Query–Value (KQV)-based techniques (137, 139, 144–146) applied across multiple cancer types. These attention-based methods have become predominant due to their superior performance. Interpretability is also essential for clinical adoption, in (144, 147), authors use the SHAP method for many cancers and colon cancer respectively. Finally, several studies (148–150) (lung, 33 types and liver cancer respectively), take advantage of many modalities integration, up to four, to be more adaptable to real world scenarios where one or more modalities are missing while providing good results.

### 6.9 Clinical translation and implementation challenges

Most multimodal AI studies in HGSOC remain retrospective proof-of-concept models with limited clinical deployment. Future translation will require integration into routine oncology workflows and interoperability with PACS, EHRs, pathology platforms, and multidisciplinary tumor board systems. Implementing these systems remains challenging due to the need for GPU resources, secure data storage, low-latency inference, maintenance costs, and specialized expertise. Multimodal pipelines must also harmonize heterogeneous radiology, pathology, molecular, and clinical data, creating major interoperability issues. In addition, non-representative training datasets may introduce and reinforce biases in model predictions. Although strategies such as adversarial debiasing, cross-modal validation, and continuous learning may improve robustness and fairness, their effectiveness in HGSOC remains uncertain (151–153). Regulatory approval pathways, medico-legal responsibility, and prospective validation requirements further complicate large-scale clinical adoption. Federated learning offers a partial solution to some of these barriers by enabling collaborative model development across institutions without centralizing sensitive patient data (154, 155) and benchmarking initiatives for federated medical AI infrastructures are beginning to emerge (156). In France, initiatives such as ConSoRe (157) illustrate the increasing integration of EHR and NLP-based infrastructures for multicentric oncology research, offering a concrete example of how real-world clinical data can be exploited at scale. Ultimately, future multimodal AI systems will need to combine predictive performance, interpretability, interoperability, and continuous clinical monitoring to become actionable clinical tools rather than isolated research prototypes.

## 7 Conclusion and outlook

This review analyzed 31 HGSOC studies through a unified multimodal pipeline taxonomy, revealing both the rapid technical evolution of the field and its persistent limitations. Several converging trends emerge from this analysis. Foundation models are reshaping feature extraction: the shift from handcrafted radiomic descriptors to large-scale pre-trained encoders such as CONCH (55), MedSAM (110) or UNI (52), reduces dependency on large annotated cohorts and opens the door to more generalizable representations across institutions (26, 27). However, evidence regarding their robustness and clinical generalizability in HGSOC remains limited, as most studies rely on retrospective monocentric cohorts with modest sample sizes. Multimodal foundation models jointly pre-trained on imaging, pathology, and genomics may represent an important future direction, although their clinical value remains to be demonstrated through prospective validation.

Concurrently, attention-based intermediate fusion is increasingly explored in multimodal oncology pipelines. While late fusion remains attractive because of its robustness to missing modalities and implementation simplicity, intermediate fusion approaches incorporating cross-modal attention mechanisms may better capture interdependencies between modalities and enable patient-specific weighting of modality contributions. Nevertheless, no clear consensus has yet emerged regarding the superiority of one fusion strategy over another in HGSOC, largely due to differences in datasets, endpoints, and evaluation protocols across studies. More generally, across other cancer types, no fusion stage or operator also appears to consistently outperform the others across all scenarios, as previously highlighted in the multimodal AI literature (158).

Then, missing modality robustness remains an open problem. Real-world clinical deployment requires models that gracefully handle incomplete data. With the exception of (27), no reviewed study treats this as a core contribution, representing a critical gap between research and clinical translation. This limitation is compounded by a fundamental trade-off rarely discussed in the reviewed literature: whether small, multimodal cohorts with heterogeneous data acquisition can achieve better generalization than larger, unimodal cohorts with standardized protocols. Given the high dimensionality of multimodal feature spaces, this remains an open empirical question that future comparative studies should address.

More broadly, the current body of evidence remains exploratory. Most reviewed studies rely on small, retrospective, monocentric cohorts, often without extensive external validation or reproducibility analyses. Omics integration is underexplored in HGSOC specifically, where multiomics datasets are scarce. Future efforts should prioritize prospective multicentric data collection combining imaging, histopathology, and molecular profiling.

Ultimately, the challenge is not purely technical but translational: models must be validated on prospective, multicentric cohorts with standardized acquisition protocols, paired with interpretability tools such as attention heatmaps, SHAP or Grad-CAM, to make predictions interpretable for clinicians. At present, multimodal AI in HGSOC should therefore be viewed as a promising but still early-stage research field rather than a clinically established paradigm.

## Glossary

HGSOC: High-Grade Serous Ovarian Cancer
CA-125: Cancer Antigen 125
HRD: Homologous Recombination Deficiency
HRP: Homologous Recombination Proficiency
BRCA: BReast CAncer DNA Repair Associated
FIGO: International Federation of Gynecology and Obstetrics
PARP: Poly (ADP-Ribose) Polymerase
TP53: Tumor Protein 53
KELIM: CA-125 Elimination Rate Constant K
ctDNA: Circulating Tumor DNA
MAF: Minor Allele Frequency
t-MAD: Tumor-informed Median Absolute Deviation
TME: Tumor MicroEnvironment
ECOG: Eastern Cooperative Oncology Group (performance status)
PFS: Progression-Free Survival
OS: Overall Survival
RFS: Recurrence-Free Survival
DFS: Disease-Free Survival
NACT: NeoAdjuvant ChemoTherapy
R0: Resection with no residual tumor (complete resection)
CT: Computed Tomography
MRI: Magnetic Resonance Imaging
PET: Positron Emission Tomography
PET/CT: Positron Emission Tomography/Computed Tomography
PET/MRI: Positron Emission Tomography/Magnetic Resonance Imaging
US: UltraSound
DWI: Diffusion-Weighted Imaging
ADC: Apparent Diffusion Coefficient
T1W: T1-Weighted
T2W: T2-Weighted
SUVmax: Maximum Standardized Uptake Value
ROI: Region Of Interest
VOI: Volume Of Interest
HU: Hounsfield Unit
ISM: Inter-Site Similarity Matrix
WSI: Whole-Slide Image
H&E: Hematoxylin and Eosin
IHC: ImmunoHistoChemistry
CLAM: Clustering-constrained Attention Multiple Instance Learning
SNV: Single Nucleotide Variation
RPPA: Reverse Phase Protein Array
DESeq2: Differential Expression Sequencing tool 2
AI: Artificial Intelligence
ML: Machine Learning
DL: Deep Learning
CNN: Convolutional Neural Network
ViT: Vision Transformer
MLP: MultiLayer Perceptron
RNN: Recurrent Neural Network
MIL: Multiple Instance Learning
LASSO: Least Absolute Shrinkage and Selection Operator
SimCLR: Simple framework for Contrastive Learning of visual Representations
GLCM: Gray-Level Co-occurrence Matrix
SHAP: SHapley Additive exPlanations
Grad-CAM: Gradient-weighted Class Activation Mapping
XGBoost: eXtreme Gradient Boosting
KQV: Key–Query–Value (attention mechanism)
FDA: Food and Drug Administration
EMA: European Medicines Agency
TCGA: The Cancer Genome Atlas
TCIA: The Cancer Imaging Archive
TCPA: The Cancer Proteome Atlas
C-index: Concordance index (Harrell’s)
KM: Kaplan–Meier
HR: Hazard Ratio
OR: Odds Ratio
AUC: Area Under the ROC Curve
ICC: Intra-Class Correlation Coefficient
ANOVA: ANalysis Of VAriance
Acc: Accuracy
Se: Sensitivity
Sp: Specificity
PPV: Positive Predictive Value
NPV: Negative Predictive Value
G-mean: Geometric Mean of Se and Sp
TPR: True Positive Rate
TNR: True Negative Rate
Dice: Dice Similarity Coefficient
IoU: Intersection over Union
IQR: Interquartile Range
RMSE: Root Mean Square Error
IDI: Integrated Discrimination Improvement
*r_s_*: Spearman’s Correlation Coefficient
*r_pb_*: Point Biserial Correlation Coefficient
VIF: Variance Inflation Factors
HL test: Hosmer–Lemeshow test
DCA: Decision Curve Analysis
NRI: Net Reclassification Improvement.
